# Effects of Culling on Mesopredator Population Dynamics

**DOI:** 10.1371/journal.pone.0058982

**Published:** 2013-03-20

**Authors:** James C. Beasley, Zachary H. Olson, William S. Beatty, Guha Dharmarajan, Olin E. Rhodes

**Affiliations:** Savannah River Ecology Laboratory, University of Georgia, Aiken, South Carolina, United States of America; Université de Sherbrooke, Canada

## Abstract

Anthropogenic changes in land use and the extirpation of apex predators have facilitated explosive growth of mesopredator populations. Consequently, many species have been subjected to extensive control throughout portions of their range due to their integral role as generalist predators and reservoirs of zoonotic disease. Yet, few studies have monitored the effects of landscape composition or configuration on the demographic or behavioral response of mesopredators to population manipulation. During 2007 we removed 382 raccoons (*Procyon lotor*) from 30 forest patches throughout a fragmented agricultural ecosystem to test hypotheses regarding the effects of habitat isolation on population recovery and role of range expansion and dispersal in patch colonization of mesopredators in heterogeneous landscapes. Patches were allowed to recolonize naturally and demographic restructuring of patches was monitored from 2008–2010 using mark-recapture. An additional 25 control patches were monitored as a baseline measure of demography. After 3 years only 40% of experimental patches had returned to pre-removal densities. This stagnant recovery was driven by low colonization rates of females, resulting in little to no within-patch recruitment. Colonizing raccoons were predominantly young males, suggesting that dispersal, rather than range expansion, was the primary mechanism driving population recovery. Contrary to our prediction, neither landscape connectivity nor measured local habitat attributes influenced colonization rates, likely due to the high dispersal capability of raccoons and limited role of range expansion in patch colonization. Although culling is commonly used to control local populations of many mesopredators, we demonstrate that such practices create severe disruptions in population demography that may be counterproductive to disease management in fragmented landscapes due to an influx of dispersing males into depopulated areas. However, given the slow repopulation rates observed in our study, localized depopulation may be effective at reducing negative ecological impacts of mesopredators in fragmented landscapes at limited spatial and temporal scales.

## Introduction

Worldwide, changing land use and the extirpation of apex predators have facilitated explosive growth in mesopredator populations [1], which in turn can have cascading effects on the dynamics of biological communities and transmission dynamics of pathogens [2], [3], [4], [5]. In particular, fragmented ecosystems often exhibit significant shifts in predator community composition as they contain insufficient habitat for the persistence of large predators while providing increased food resources for generalist species. Thus, human-modified ecosystems generally support high densities of mesopredator populations due to relaxation of top down and bottom up regulatory mechanisms, enhancing pressures on populations of prey species beyond the direct effects of fragmentation and increasing the potential for disease outbreaks [6], [7].

Common disease control practices for mesopredators include localized culling, landscape or region-wide baiting regimes, and trap-vaccinate-release programs [8], [9], [10], [11]. In particular, culling is often used in response to disease outbreaks of serious zoonotic or economic concern (e.g., bovine tuberculosis, rabies). In addition to disease mitigation, population reduction of mesopredators also is widely used to alleviate predation pressure on a wide range of taxa [12], [13], [14], which may have unintended consequences of altering disease transmission dynamics in remnant predator populations. Despite the widespread use of culling in the management of mesopredators, the long-term efficacy of this method for mitigating spread of disease is poorly understood. Moreover, density reductions alone may not reduce prevalence of disease due to behavioral changes in colonizing individuals [10], [15], [16]. Similarly, culling may alter the social or genetic structure of colonizing populations, potentially altering contact rates and disease exposure risk [17].

The efficacy of culling at reducing prevalence of infectious disease also may be influenced by the speed and mechanism of population reestablishment (i.e. natal dispersal vs. home range expansion) due to intersexual and age-specific differences in communicability of diseases [18], [19]. In mammals, short-term population recovery may occur through natal dispersal (often by males; [20]), or by migration of adults into depopulated areas due to reduced resource competition (vacuum effect; [21], [22]). Such colonization scenarios can occur over different time scales and produce vastly different demographic and disease profiles within recolonized populations. However, the contributions of natal dispersal and home range shifting in population recolonization is unclear for many species, and may differ spatially due to differences in the distribution and quality of habitats within landscapes [23], [24].

Of all North American mesopredators, raccoons have exhibited one of the most positive responses to land use change and the extirpation of apex predators over the last few centuries [4]. Increased raccoon densities have been linked to cascading effects on local biological communities through increased predation on a diverse array of taxa [6], [2], [25], [26] and increased transmission of infectious diseases [9], [27]. Moreover, as raccoons are the primary reservoirs of rabies in North America, their increasing numbers represent acute threats to human and livestock health [8], [11].

Due to their overwhelming ecological and economic impacts (e.g., rabies costs exceed $400 million in the U.S. annually; [28]), raccoons have been subjected to extensive control throughout much of their range. Targeted culling, in particular, is frequently used in raccoon control, especially for localized management (e.g., [13], [25]). However, few studies have monitored demographic or behavioral responses of raccoons to population reduction [29], [30], and no studies have investigated population recovery in this species as a function of landscape composition or configuration.

The aim of this paper was to evaluate the efficacy of targeted mesopredator removal programs within fragmented landscapes. To achieve this goal, we performed a replicated raccoon removal experiment and contrasted demographic parameters of recolonizing raccoon populations against pre-removal estimates. We then determined whether the rate of population recovery was influenced by habitat fragmentation or spatial variance in local patch attributes. In addition, we incorporated long-term data for 25 control patches as a baseline measure of temporal patterns in raccoon demography during our experiment. Using a highly replicated experimental framework (30 experimental patches), we tested the following hypotheses regarding the population recovery of raccoons following complete depopulation: 1) patch colonization is dominated by natal dispersal rather than home range shifting by adults from neighboring patches (i.e. vacuum effect) due to the small home range sizes of adult raccoons in fragmented agricultural ecosystems [31]; 2) recolonizing populations exhibit increased apparent survival relative to control populations due to reduced competition for resources and niche availability; 3) patches rapidly reestablish (≤3 years) to pre-removal demographic levels within agricultural ecosystems given the high densities of raccoons that exist in these ecosystems and behavioral plasticity of this species; but 4) recolonization rates differ among patches as a function of landscape connectivity and local patch quality. The implications of this research are then discussed in the context of current mesopredator management programs, particularly those aiming to limit the spread of infectious diseases in fragmented ecosystems.

## Materials and Methods

### Ethics Statement

All raccoons were captured using box livetraps and handled or euthanized in accordance with American Society of Mammalogist guidelines [32] and as authorized under Purdue Animal Care and Use Committee protocols 07–018 and 01–079. All efforts were made to minimize animal suffering. Collection permits for this research were obtained from the Indiana Department of Natural Resources.

### Study Area

**Figure 1:**
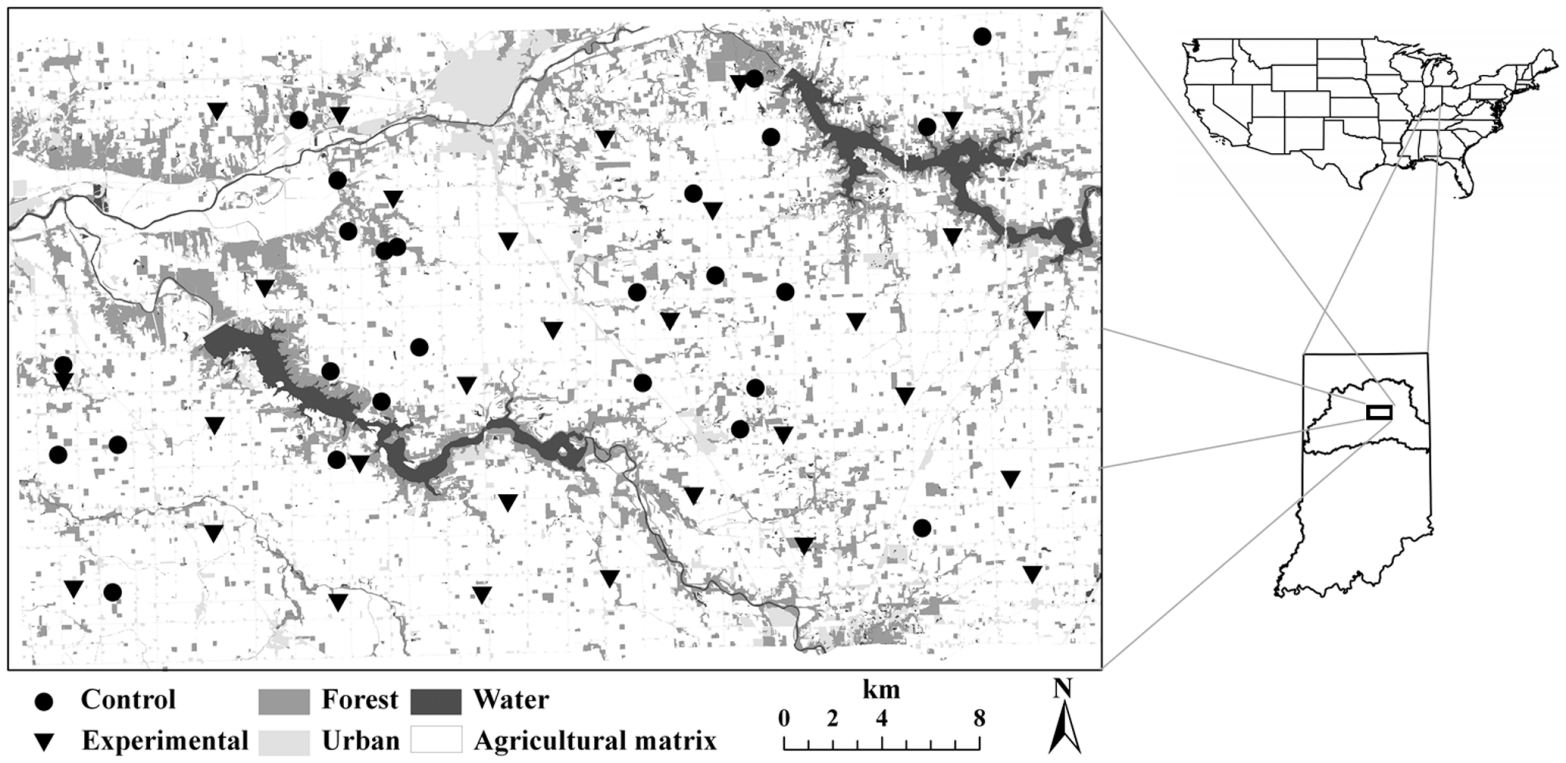
Spatial distribution of experimental (*n* = 30) and control (*n* = 25) forest patches used in this study throughout north-central Indiana, USA.

This study was conducted within a 1,165 km^2^ area of the Upper Wabash River Basin (UWB) in northcentral Indiana, USA (Figure 1). Approximately 96% of the UWB was privately owned, 66% of which was in agricultural production. The primary agricultural crops in the UWB were corn and soybeans with small interspersed fields of hay and small grains. All contiguous forest tracts were confined to major drainages where frequent flooding or steep topography made the land unsuitable for crop production. The remaining native forests were highly fragmented and dominated by patches <5 ha. Although the majority of the landscape was comprised of agricultural matrix, almost all raccoon activity was restricted to forested habitats or forest-agricultural interfaces [33], [34]. Thus, raccoon removal efforts in this experiment were limited to forested habitats.

We selected 30 privately owned forest patches to examine population recovery of raccoons following depopulation. Patches were selected based on a combination of size and isolation criteria. We estimated patch size using ArcMap 9.2 and calculated an index of isolation for each forest patch using patch-based metrics in FRAGSTATS based on a 1-km search radius with an eight-neighbor rule (ver. 3.3, PROX function; [35]). Only patches between 3 and 12 ha were incorporated into the patch selection procedure to ensure complete depopulation would be feasible and to minimize variance in recolonization rates as a function of patch size. Based on the distribution of isolation values in our study area, we selected 10 patches within each of three ranges of isolation metrics that represented the dominant landscape configurations in the UWB (isolated = highly isolated, intermediate = clusters of patches varying in proximity to one another, and connected = mainland-island systems). Selected patches were uniformly distributed throughout the study area, and no two patches were selected within 3.8 km of one another: a distance twice the diameter of the maximum raccoon home range size in this landscape [31].

In addition to our 30 experimental patches, we used mark-recapture data for 25 control patches collected from 2007–2010 within our study area as a baseline measure of temporal patterns in demography during the course of this experiment [7]. Control patches were distributed uniformly throughout the UWB and thus, occurred across the same range of habitat metrics as experimental patches (Figure 1).

### Removal

From March to June of 2007, raccoons were captured using box live traps (Tomahawk Live Trap Co., Tomahawk, Wisconsin, USA) baited with cat food, sardines, or marshmallows. We collected tissue, blood, and standard morphological and demographic data from euthanized animals which were subsequently necropsied to collect additional tissue samples for further analyses. Traps were placed strategically (e.g., along fallen logs and streams, near latrines, at the base of den trees) throughout selected forest patches at a density of ∼4 traps/ha (maximum of 30 traps per patch) and pre-baited for 1–3 nights. Subsequently, traps were opened and maintained for a minimum of 14 days or until no signs of raccoon activity (e.g., tracks, trap disturbances) were observed within patches for 4 consecutive days. At this point we considered patches to be depopulated of raccoons. Raccoons in our study landscape maintain small home ranges presumably due to the proximity of food and denning resources (*
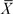
* = 73 ha; [31], [34]) and are highly susceptible to trapping during the spring prior to the emergence of agricultural food resources [7]. Thus, trapping was capped at 25 days as this was believed to be a sufficient time period to remove all resident raccoons. Our decision to cap trapping efforts at 25 days was later supported by the fact that 71% of removed raccoons were captured before day 10 and 94% were captured on or before day 20.

Following euthanasia we collected weight and gender for each individual. In addition, we collected tissue for genetic analysis and a tooth (PM1) which was aged to the nearest year via cementum analysis (Matson's Laboratory LLC, Milltown, Montana, USA).

### Measurements of recolonization

To determine time until initial recolonization we placed a single digital remote camera (model STC-WD1; Stealth Cam LLC, Grand Prairie, Texas, USA) baited with fish oil or ear corn covered with peanut butter in each patch at the conclusion of trapping. Cameras were placed along streams or other locations likely to capture images of raccoons utilizing experimental patches and re-baited as necessary until evidence of recolonization was observed. In addition, raccoons captured or observed on camera during concurrent experiments within experimental patches (e.g., small mammal trapping [36] or scavenging trials [37]) were used to supplement camera data to confirm initial recolonization. A forest patch was considered recolonized upon detection of a raccoon.

From 2008–2010, experimental patches were trapped annually during spring (March–June) to estimate abundance and demographic parameters for each recolonizing population. Control patches were trapped concurrently with experimental patches and mark-recapture trapping and handling procedures employed in all patches were identical to those described in [7]. Briefly, traps were placed in a grid (50-m spacing) within forest patches and pre-baited for 1–3 nights. The total number of traps per grid varied with patch size, with a maximum of 30 traps placed in any single forest patch. Following the pre-baiting period traps were opened and maintained for 10 consecutive nights. All captured raccoons were tagged (Monel #3, National Band and Tag Company, Newport, KY) and we collected blood, tissue, and standard morphological and demographic data. In addition, we collected a tooth (PM1) to determine age for recolonizing individuals; age was determined in control patches based on patterns of tooth wear and individuals were classified as juveniles (0–1), yearlings (1–2), and adults (≥3) [38].

### Abundance

In removal patches, for 2007 both overall and female-only abundance were calculated as the total number of individuals removed within each patch because our trapping protocol was believed to represent a complete census of the resident population. In subsequent years we estimated raccoon abundance (overall and female-only) following the methods outlined in [7]. Briefly, we modeled abundance using the Huggins closed capture-recapture modeling procedure in Program MARK [39]. To overcome problems associated with low numbers of individuals per patch, we modeled the combined data from all 30 patches each year to obtain parsimonious models of capture and recapture parameters for the combined data set, but obtained patch specific estimates of N by treating each patch as a disparate attribute group in MARK [7]. We developed separate models for each of the 3 years to minimize violations of the closure assumption. Both gender and age of raccoons were incorporated as covariates in overall abundance models and raccoon age was included as a covariate in female-only models. We tested a suite of 39 models incorporating these covariates as well as constant and time specific capture and recapture parameters that we felt could contribute to variation in raccoon capture probabilities. Model fit was evaluated using a bias-corrected version of Akaike' information criterion (AIC_c_) and we used model averaging to determine final population sizes for all models deviating ≤4 AIC_c_ units from one another [40].

Using MARK-derived population sizes we estimated density by overlaying a buffer equal to the average raccoon home range size in our study area (73 ha; [31]) centered on each trapping grid. Buffers were assigned to approximate our effective trapping area by accounting for raccoon movements.

### Habitat Attributes

Landscape-level habitat attributes were estimated for each forest patch using a GIS database developed from 1998 U.S. Geological Survey digital orthophotos (DOQs) of 1-m resolution (details of habitat delineations are provided in [7]. For each patch we estimated size, as well as total area (ha) of forest, agriculture, grassland, developed, wetland (including ponds/lakes), and stream (length) habitats within a 92 ha buffer of the centroid of the patch using ArcMap 9.2 (Table S1). We selected a 92 ha buffer to account for movement behavior of raccoons as this represents an area equal to the average home range size of males (which exceed those of females) in our study area [31].

Local habitat attributes reflecting vegetative community composition and availability of denning resources were collected through fine-scale, field-based vegetation and den tree surveys, respectively. Specific details of survey methodology are described in [7] and a list of habitat variables incorporated into our analyses is provided in Table S1.

### Statistical Analyses

Landscape-level changes in raccoon density during the course of this study (e.g., due to a distemper outbreak) could have introduced a source of bias into this experiment. To determine if the overall raccoon population within our study landscape fluctuated during the course of this study we used Analysis of Variance (ANOVA) to compare temporal differences in overall population density and female abundance (i.e. number captured) based on data from our 25 control patches collected during 2007–2010. Annual patch-specific estimates of density and number of females were included as dependent variables and year (2007–2010) served as the independent variable in both analyses. We also used ANOVA to evaluate variance in time to recolonization of experimental patches across the three levels of forest patch isolation. All statistical analyses were performed using SAS ver. 9.1 (SAS Institute, Cary, NC).

To evaluate temporal recovery of raccoon densities and female abundance relative to time since removal (years) and patch isolation, we used linear mixed models with repeated measures. We developed separate models for raccoon density and female abundance using a heterogeneous compound symmetry covariance structure. Kolmogorov-Smirnov tests indicated deviations from normality for female abundance, thus estimates of female abundance were square-root transformed. Year, isolation class, and the year*isolation interaction were included in both models as fixed effects, patch as a random effect, and year as the repeated measure. For each year following depopulation, we determined whether the overall suite of experimental patches had recovered to pre-removal levels using Tukey multiple comparisons tests for models in which the main effect year was significant.

We used ANOVA to determine whether age structure (all individuals within experimental patches combined within a given year) of colonizing raccoons differed from the pre-removal age structure, and to identify whether changes in age structure were influenced by differences in isolation among experimental patches. Year, isolation class, and the year*isolation interaction were included as independent variables. Significant main effects were explored using Tukey multiple comparisons tests. Kolmogorov-Smirnov tests indicated that residuals from this analysis were non-normally distributed; therefore we square-root transformed raccoon age. Transformed variables were back-transformed prior to inclusion in figures.

For 2008 and 2009 we constructed an index of apparent survival for both control and experimental patches based on the proportion of tagged individuals (overall and separately for each sex) recaptured between 2008–2009 and 2009–2010, respectively. Prior to this analysis we used genetic data to identify individuals that had lost tags and been reassigned as new individuals during a subsequent year (*n* = 17) to eliminate bias in survival estimates due to tag loss (See [7] for methodological details). If individuals were not captured in the year after their initial capture, but were captured again in the subsequent year, they were assumed present during the intervening year. To test the hypothesis that colonizing raccoons exhibit higher apparent survival than individuals in control patches due to relaxation of density-dependent regulatory mechanisms, we used exact binomial tests to evaluate whether apparent survival differed between experimental and control patches during 2009 and 2010 by sex and for all individuals combined.

Because apparent survival confounds mortality with emigration, we also explored the distribution of new captures across 10-day mark-recapture sampling periods to quantify raccoon residency within experimental patches and ensure estimates of apparent survival were not biased due to emigration by colonizing individuals. Raccoons are highly susceptible to trapping during spring when agricultural food resources are limited and thus likely to enter traps [7] so distributions of new captures across 10-day trapping periods should provide useful indices of residency for individuals within patches (i.e. most residents should be captured in the first few days; see Results). Deviance in the distribution of captures between control and experimental patches provides important insights into whether raccoons were true residents or temporary immigrants exploring depopulated patches. We used log-linear analysis (PROC CATMOD) to examine differences in the distributions of new captures across 10-day sampling periods among years. If a significant treatment*fate (i.e. year*trap day) interaction was observed, indicating that distributions of initial captures across 10-day sampling periods differed among years, we repeated our analysis individually for each year of post-removal monitoring to determine which years exhibited distributions of captures differing from that observed in control patches.

Finally, we evaluated the influence of habitat attributes on population recovery using generalized linear models. We developed separate models for density and female abundance and considered patches recolonized if they attained pre-removal levels in the final year of sampling (2010) or in at least two of the three years of post-removal sampling. Pearson correlation tests indicated that multicollinearity (≥0.50) existed among many of our habitat variables. Thus, we conducted principal components analyses (PCA) with varimax rotation on the correlation matrix of habitat variables to reduce dimensionality. We performed separate PCA analyses for local and landscape-level habitat attributes. Components from both analyses with eigenvalues >1 were incorporated into logistic regression models for both overall density and female abundance (0 = not recovered, 1 = recovered; see above) to identify habitat features explaining significant proportions of the variance in the demographic recovery of raccoon populations.

## Results

### Raccoon Removal

Between 31 March and 18 June 2007 we removed 382 raccoons ≥1 year old. Numbers of raccoons removed ranged from 1 to 33 among patches (*
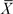
* = 12.73, SD = 7.98; Table S2). Extensive variability in sex ratio was observed among experimental patches, with patches ranging from 0 to 100% female.

### Raccoon Recolonization

Within control patches, neither raccoon density nor the number of females captured differed across the 4 years of this study (*F*_3,96_ = 0.48, *p* = 0.70; *F*_3,96_ = 1.05, *p* = 0.38, respectively). Subsequent to depopulation, raccoons quickly colonized vacant patches. Time to recolonization varied extensively among patches (range: 15–135 days, *
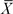
* = 59), but was not influenced by patch isolation (*F*_2,27_ = 1.59, *p* = 0.22). Although raccoons were detected in all experimental patches within five months following depopulation, none were captured in two experimental patches during mark-recapture trapping in 2008 (range 0–12, *
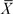
* = 5.4). Raccoons were captured in all 30 experimental patches in 2009 (range 2–17, *
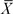
* = 7.9) and 2010 (range 1–13, *
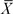
* = 6.1). Interestingly, we did not capture any females in 10 experimental patches in 2008 (range 0–5, *
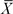
* = 1.5) and 8 patches in 2010 (range 0–7, *
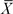
* = 2.1), although females were captured in all patches in 2009 (range 1–9, *
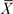
* = 3.0).

Model selection results across the 3 years of mark-recapture sampling produced 4–8 competitive models (ΔAIC = 4) for estimates of overall raccoon abundance and 2–9 competitive models for estimates of female abundance. Based on model averaged estimates of these models, raccoon abundance ranged from 0–17 among experimental patches in 2008 (*
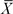
* = 3.0, SD = 4.2), 2–18 in 2009 (*
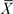
* = 8.7, SD = 4.3), and 1–16 in 2010 (*
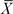
* = 7.4, SD = 4.1). Model averaged estimates of female abundance ranged from 0–6 in 2008 (*
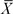
* = 1.9, SD = 1.9), 1–10 in 2009, (*
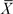
* = 3.4, SD = 2.3), and 0–8 in 2010 (*
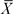
* = 2.4, SD = 2.1).

**Figure 2:**
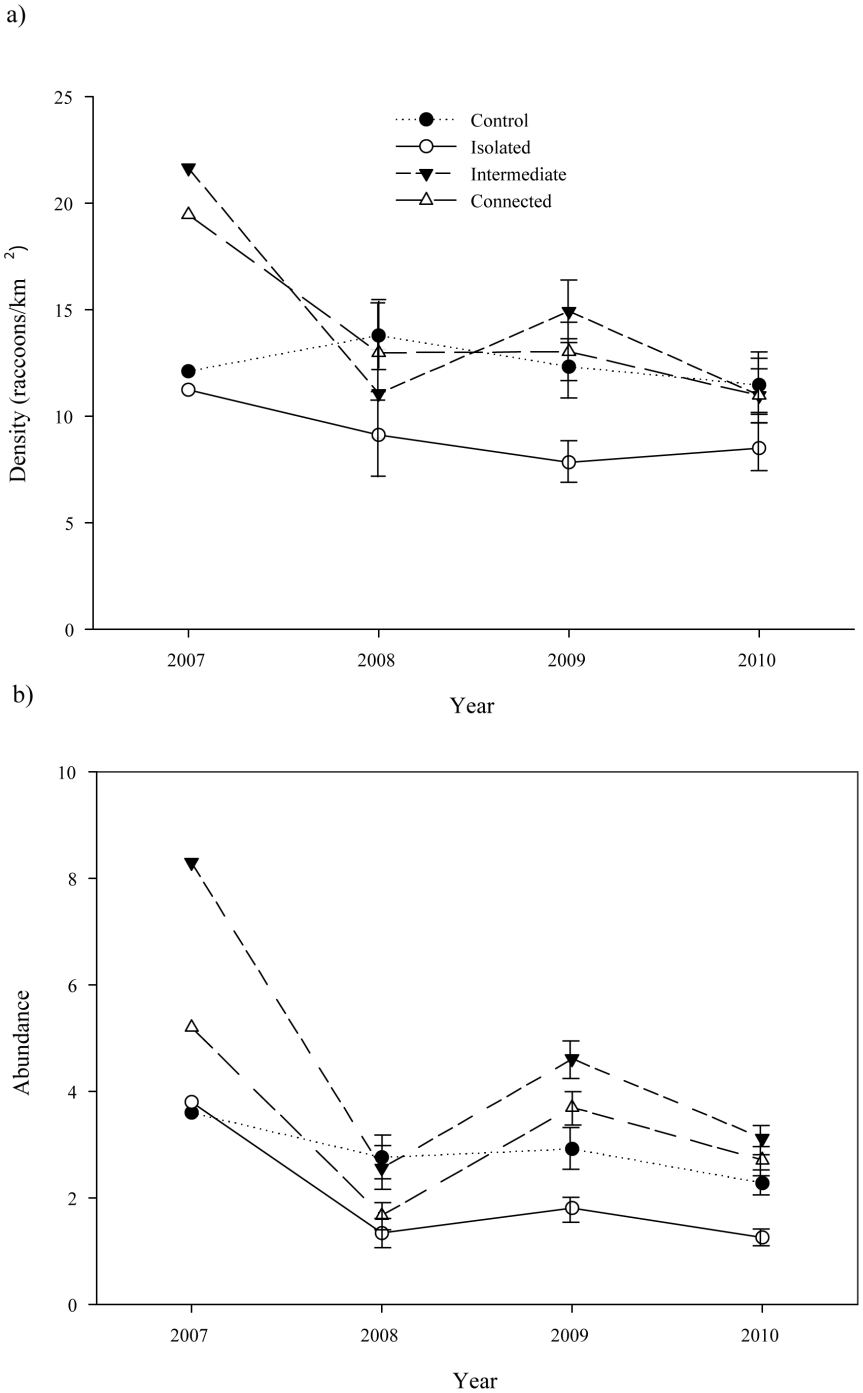
Estimated a) density and b) female abundance of raccoons averaged across 25 control and 30 experimental (divided among three patch isolation categories: isolated, intermediate, and connected) forest patches monitored from 2007–2010 in north-central Indiana, USA. Experimental patches were completely depopulated in 2007 and allowed to recolonize naturally.

Within experimental patches raccoon densities differed among years (*F*_3,81_ = 4.54, *p* = 0.005) and between isolation classes (*F*_2,27_ = 5.31, *p* = 0.011), but the interaction between year and isolation was not significant (*F*_6,81_ = 0.94, *p* = 0.470). Despite rapid colonization of experimental patches, less than 40% had recovered to pre-removal densities (i.e. within 25% of original estimate) in any of the three years after depopulation (2008 – 40%, 2009 – 33%, 2010 – 37%). Tukey post-hoc tests adjusted for multiple comparisons revealed that after three years, overall mean patch densities had not recovered to pre-removal levels (*p*<0.05 for all comparisons between 2007 and 2008–2010; Figure 2a), failing to support our hypothesis that patches would recover to pre-removal densities within 3 years. Following initial estimates of densities for recolonizing individuals in 2008 (*
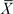
* = 11.1, SD = 5.7), experimental patches appeared to stabilize and did not increase in density over the next two years [2009 – (*
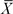
* = 11.9, SD = 5.9), 2010 – (*
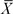
* = 10.1, SD = 5.7); *p*>0.05 for comparisons between 2008 and 2010; Figure 2a].

Recolonization in this study was more limited for females than males, as on average female abundance had recovered to only 32% of pre-removal levels one year following removal, and we caught no females in eight patches (27%) three years after depopulation. Female abundance differed among years (*F*_3,81_ = 15.31, *p*<0.0001) and isolation classes (*F*_2,27_ = 6.12, *p* = 0.006), although the interaction of year and isolation was not significant (*F*_6,81_ = 0.5, *p* = 0.810), indicating patterns of recovery were similar among patches regardless of isolation class. As with overall density, female abundance was significantly less than pre-removal levels during each post-removal sampling period (*p*<0.05 for comparisons between 2007 and 2008–2010; Figure 2b). However, unlike overall density, female abundance increased from 2008 to 2009 (*t* = −4.23, *p*<0.001) and subsequently declined from 2009 to 2010 (*t* = 3.07, *p* = 0.015; Figure 2b).

The overall age structure of raccoons in experimental patches changed in response to removal (*F*_3,952_ = 13.64, *p*<0.001). During all post-removal years, experimental populations were younger than pre-removal populations (2007–2008: *t* = 5.46, *p*<0.0001; 2007–2009: *t* = 4.61, *p*<0.0001; 2007–2010: *t* = 4.03, *p* = 0.0004), and age structures did not differ among post-removal years (2008–2010, *p*>0.05). Mean age of raccoons captured in experimental patches was 3.8 during 2007 (removal year; SD = 2.3), 2.8 in 2008 (SD = 2.0), 3.1 in 2009 (SD = 2.3), and 3.1 in 2010 (SD = 2.2). Overall population age structure also differed among isolation classes (*F*_2,952_ = 5.52, *p* = 0.004), with isolated patches exhibiting a younger age structure (*
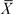
* = 3.0, SD = 2.4) compared to both intermediately (*
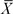
* = 3.4, SD = 2.1) and highly connected (*
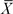
* = 3.5, SD = 2.4) patches (*p*<0.05).

Our prediction that colonizing raccoons would exhibit increased rates of apparent survival due to reduced competition for resources received mixed support. Individuals initially colonizing experimental patches in 2008 and 2009 exhibited lower apparent survival than raccoons in control patches (*p* = 0.03, *p* = 0.04, respectively). Specifically, apparent survival of raccoons in experimental patches was 0.30 in 2008 and 0.28 in 2009 compared to 0.39 and 0.35 in control patches, respectively. However, during both years reduced apparent survival in experimental patches was driven by a reduction in apparent survival of colonizing males (2008: 0.19 vs. 0.36, *p*<0.001; 2009: 0.28 vs. 0.38, *p* = 0.001). Colonizing females actually exhibited higher survival rates than females in control patches in 2008 (0.61 vs. 0.44, *p* = 0.04), and similar survival rates in 2009 (0.30 vs. 0.30, *p* = 0.94).

Although males exhibited reduced apparent survival in experimental patches during 2008 and 2009, this likely reflects increased emigration rather than an actual reduction in true survival. Log-linear analyses revealed that distributions of new captures across 10-day sampling periods differed among years in experimental patches (likelihood ratio *χ* ^2^_27_ = 50.91, *p* = 0.004). Exploration of this pattern indicated that differences in capture history distributions existed during 2008 and 2009 relative to distributions observed within control patches (likelihood ratio *χ* ^2^_9_ = 19.63, *p* = 0.02;likelihood ratio *χ* ^2^_9_ = 17.87, *p* = 0.04, respectively), but by 2010 experimental populations had stabilized and were primarily comprised of residents (likelihood ratio *χ* ^2^_9_ = 9.81, *p* = 0.37). We suspect that many raccoons captured in 2008 and 2009 were not true residents, but rather were dispersing raccoons or individuals from neighboring patches exploring habitats at the periphery of their home range. For example, across 1,957 individuals captured in control patches, 35% were initially captured on the first day of the 10-day trapping period and over 50% were captured in the first two days. In contrast, of the 163 raccoons captured in experimental patches in 2008, only 24% were initially captured on the first day of trapping and it took four days to capture 50%.

Principal component analysis extracted 2 landscape and 4 local components (eigenvalues >1) explaining 68% and 67% of the variance in the original habitat variables, respectively. The first landscape component was dominated by positive factor loadings for the availability of forest and grassland habitats, and a negative loading for the availability of agriculture, whereas the second component included a positive factor loading for forest patch size and a negative loading for the availability of developed habitats. Local components were characterized by positive factor loadings for overall basal area and understory stand density (Prin 1), positive loadings for stream length, availability of forbs, and availability of soft mast (Prin 2), a positive loading for shrub density and negative loading for plant diversity (Prin 3), and a negative loading for the availability of wetlands (Prin 4). However, none of these components explained a significant portion of the variance in recolonization rates among experimental patches for either overall density or female abundance (*p*>0.05), failing to support our hypothesis that recolonization rates would be positively correlated with landscape connectivity and habitat attributes associated with patch quality (e.g. water availability, den trees; [7]).

## Discussion

Although culling is widely used to mitigate the spread of disease in mesopredators, such perturbations can cause severe disruptions in the movement behavior and population dynamics of remnant populations and in some cases may even facilitate dissemination of disease [10], [15], [16]. Such changes in behavior and population structure in response to removal may be further enhanced in landscapes that have been highly modified by human landuse due to unique spatial constraints imposed on organisms in these systems. In this study we demonstrate a significant shift in the density, sex ratio, and age structure of recolonizing mesopredator populations following complete depopulation in a highly fragmented agricultural ecosystem. However, neither the rate nor demographic patterns of patch colonization were influenced by the composition nor configuration of landscape attributes. These results build upon the growing body of evidence that population manipulation alone may be insufficient to control the spread of infectious disease in mesopredators [10], [15], [16], [18].

Despite a rapid initial colonization of vacant habitat patches, after three years only 40% of experimental patches had recovered to pre-removal densities. This stagnant recovery appeared to be driven by limited colonization of patches by females, which likely resulted in limited within-patch recruitment [41]. For example, three years after depopulation no females were captured in 27% of experimental patches and only 20% had achieved pre-removal numbers of females. However, females that initially colonized vacant patches exhibited increased apparent survival relative to females in control patches, likely in response to reduced competition for denning resources [7] and increased niche availability. Limited colonization of patches by females in our study likely reflects the inherent propensity of females to remain within their natal patch, as dispersal in many mammalian mesopredators is male biased, with females exhibiting high fidelity to their natal range [42], [43]. Consequently, it may take several years for raccoon populations to completely reestablish if within-patch reproduction is the primary mechanism contributing to local recruitment of females.

In addition to altering the density and sex ratio of local populations, our experimental perturbation also shifted the age structure towards younger age classes. Shifts in age structure were driven by extensive colonization of patches by 1–2 year old males, supporting our hypothesis that natal dispersal would be the primary mechanism of patch recolonization versus home range shifting by adults (i.e., vacuum effect). However, colonizing adults up to 12 years old were captured in experimental patches, suggesting some range expansion or shifting did occur. Among mesopredators the roles of range shifting and natal dispersal in patch recolonization appear to vary as a function of the size of the area depopulated, landscape characteristics, and behavioral attributes of individual species [18], [22], [44]. Thus, the efficacy of culling to mitigate spread of disease likely varies among species and across landscapes. The spatial extent of population reduction also may alter recovery rates as partial reductions (particularly of females) may facilitate more rapid recovery due to relaxation of density-dependent effects on population dynamics [30].

Despite the rapid influx of individuals into depopulated patches, the distribution of new captures across our 10-day sampling period suggested that stable, resident populations were not established until three years after manipulation. This is further supported by the reduced apparent survival of males from 2008–2009 and 2009–2010 in experimental patches, which likely reflected reduced site fidelity rather than reduced survival. Thus, while populations appeared to rapidly recover to >60% of their pre-removal densities, estimates for 2008 and 2009 undoubtedly overestimated the true size of recolonizing populations as many of these individuals probably were temporary immigrants. Given the tendency for males to establish territories in locations that will maximize their reproductive success, limited site fidelity of colonizing males probably resulted from the absence or low abundance of females within experimental patches [45].

Interestingly, population recovery in this study was not influenced by landscape connectivity or local habitat attributes. This is surprising given that movement behavior and local population dynamics of raccoons vary extensively throughout fragmented ecosystems in response to spatial variation in connectivity and local patch characteristics [7], [34]. Average dispersal distances in raccoons generally exceed 5 km and dispersal does not appear to be hindered by habitat continuity [43], [46], but see [47]. Thus, we suspect that dominance of patch colonization by natal dispersers may have masked any effects of patch connectivity or quality on recolonization rates. Consequently, landscape variables do not appear to be informative in the development of metapopulation models for this species in agricultural ecosystems [23], at least at the spatial scale of this study.

Due to the high densities of raccoons that agricultural ecosystems support and general behavioral plasticity of this species, the low levels of recovery observed in this study were surprising. However, in control patches neither overall density nor female abundance differed over the course of our experiment, suggesting that observed repopulation patterns likely reflect natural patterns of population establishment in agricultural ecosystems. Moreover, our results support previous research evaluating population recovery of mesopredators in heterogeneous landscapes [22], [48], [49]. Given the demographic patterns of population recovery we observed, it appears that emergent properties of fragmented ecosystems may facilitate the efficacy of culling operations by minimizing female recruitment. In particular, the propensity for females to remain within their natal patch may be magnified for populations persisting in heterogeneous landscapes due to increased challenges associated with dispersal in these environments.

Although epidemiological impacts were not explicitly tested in this study, the demographic changes we noted in recolonizing raccoon populations may have strong implications for disease transmission dynamics in human-modified landscapes [18], [49]. For example, rabies [50] and *Baylisascaris* [19] infections are highest in young raccoons. Thus a shift in age structure towards younger individuals, as was observed in our study, is likely to facilitate the spread of these diseases throughout the landscape [10], [15], [51]. Changes in sex ratio also may influence disease transmission dynamics as exposure may differ between sexes due to differences in life history characteristics [19] or behavior [10], [15], [17], [52]. In particular, the lack of females within depopulated patches could encourage male home range expansion during the breeding season, increasing the risk of disease spread [15], [18]. Ultimately however, the impact of altered population demography on disease prevalence is likely to vary among diseases depending on the transmission dynamics of particular pathogens, and future empirical studies are needed to quantify the effects of density manipulations on the spread of rabies and other zoonotic diseases.

Collectively, our results suggest the efficacy of mesopredator removal programs in fragmented landscapes may vary depending on specific management objectives. For programs aiming to reduce nest predation or control locally abundant populations, it appears depopulation may be an effective tool for maintaining reduced mesopredator populations at small spatial scales. Given that elevated mesopredator densities in fragmented ecosystems is one of the proximate mechanisms contributing to global declines of many avian species [6], [53], [54], removal operations resulting in population reductions may have cascading benefits throughout ecosystems [4], [5]. However, such benefits may come at the expense of increased risk of zoonotic disease dissemination due to an influx of juveniles into depopulated areas. Thus, future studies are needed to evaluate the efficacy of integrating localized culling operations within the framework of existing trap-vaccinate-release, contraceptive, and/or vaccine baiting regimes in the management of rabies and other zoonoses.

## Supporting Information

Table S1 **Description of all habitat variables measured to examine the influence of habitat characteristics on raccoon population recovery in northcentral Indiana, USA, 2007–2010.**
(DOCX)

Table S2 **Summary information for each of the 30 experimental forest patches used in this study which were distributed throughout the upper Wabash river basin in northern Indiana, USA.** Patches were depopulated of raccoons in 2007 and then allowed to naturally recolonize. Experimental patches were assigned to 3 isolation classes (estimated using the PROX function in FRAGSTATS) based on the distribution of isolation values observed in our study area: 1) highly isolated patches, 2) intermediate—clusters of patches varying in proximity to one another, and 3) connected—mainland-island systems). Density estimates for each year are based on population sizes estimated using the Huggins closed capture-recapture modeling procedure in Program MARK, incorporating a buffer encompassing an area equal to the average raccoon home range size in our study area (73 ha) to account for raccoon movements. Estimates of female abundance were derived using the Huggins closed capture-recapture modeling procedure in Program MARK.
(DOCX)
