# Supplementary material for: Effects of Culling on Mesopredator Population Dynamics
Source: PLoS One. 2013 Mar 20;8(3):e58982. doi: 10.1371/journal.pone.0058982 (PMC3604110; doi:10.1371/journal.pone.0058982)
Supplement: Table S1 — Description of all habitat variables measured to examine the influence of habitat characteristics on raccoon population recovery in northcentral Indiana, USA, 2007–2010. (DOCX) [file pone.0058982.s001.docx]

Table S1.

| Acronym | Description |
| --- | --- |
| PSIZE | Forest patch area (ha) |
| PROX | Index of patch isolation calculated using the proximity function in FRAGSTATS |
| FORBUFF | Amount of forest (ha) within a home range buffer surrounding patches |
| URBBUFF | Amount of developed (ha) within a home range buffer surrounding patches |
| AGBBUFF | Amount of agriculture (ha) within a home range buffer surrounding patches |
| GRASBUFF | Amount of grassland (ha) within a home range buffer surrounding patches |
| WETLAND | Area of wetlands (ha) within patches |
| STREAM | Length of streams (m) within a home range buffer surrounding patches |
| FORBS | Density of forbs (plants/m^2^) |
| SOFTMAST | Density of softmast producing species including understory *Acer spp*.(plants/ha) |
| GRASS | Average % coverage grass spp. (Poacea). |
| BAD | Basal area of overstory trees |
| DIVERSITY | Shannon’s diversity index of overstory, understory and herbaceous plant composition |
| TREECAV | Density of potential den tree cavities (tree cavities/ha) |
| TREES | Density (trees/ha) of all trees >25.6 cm dbh |
| SHRUB | Density of woody stems <25.6 cm DBH (stems/ha) |
