# Supplementary material for: Effects of Culling on Mesopredator Population Dynamics
Source: PLoS One. 2013 Mar 20;8(3):e58982. doi: 10.1371/journal.pone.0058982 (PMC3604110; doi:10.1371/journal.pone.0058982)
Supplement: Table S2 — Summary information for each of the 30 experimental forest patches used in this study which were distributed throughout the upper Wabash river basin in northern Indiana, USA. Patches were depopulated of raccoons in 2007 and then allowed to naturally recolonize. Experimental patches were assigned to 3 isolation classes (estimated using the PROX function in FRAGSTATS) based on the distribution of isolation values observed in our study area: 1) highly isolated patches, 2) intermediate—clusters of patches varying in proximity to one another, and 3) connected—mainland-island systems). Density estimates for each year are based on population sizes estimated using the Huggins closed capture-recapture modeling procedure in Program MARK, incorporating a buffer encompassing an area equal to the average raccoon home range size in our study area (73 ha) to account for raccoon movements. Estimates of female abundance were derived using the Huggins closed capture-recapture modeling procedure in Program MARK. (DOCX) [file pone.0058982.s002.docx]

Table S2.

| Patch | Patch size (ha) | Isolation class | Density (2007) | Female abundance (2007) | Density (2008) | Female abundance (2008) | Density (2009) | Female abundance (2009) | Density (2010) | Female abundance (2010) |
| --- | --- | --- | --- | --- | --- | --- | --- | --- | --- | --- |
| AGD | 3.97 | 1 | 16.44 | 4 | 8.16 | 1.11 | 7.58 | 1.10 | 10.01 | 3.52 |
| AND | 6.21 | 1 | 13.70 | 3 | 0.00 | 0.00 | 7.58 | 1.16 | 3.33 | 0.00 |
| CLN | 5.57 | 1 | 23.29 | 11 | 14.48 | 2.21 | 12.09 | 2.19 | 9.99 | 1.11 |
| HWT | 3.36 | 1 | 1.37 | 1 | 8.33 | 0.00 | 4.51 | 1.16 | 5.01 | 0.00 |
| OVN | 5.80 | 1 | 21.92 | 9 | 8.40 | 1.11 | 9.14 | 1.16 | 13.30 | 3.39 |
| PLN | 11.78 | 1 | 12.33 | 5 | 17.90 | 4.48 | 6.05 | 1.10 | 5.01 | 1.17 |
| RDR | 3.72 | 1 | 5.48 | 1 | 0.00 | 0.00 | 2.96 | 1.16 | 8.34 | 0.00 |
| WAB | 3.74 | 1 | 4.11 | 0 | 8.58 | 0.00 | 10.55 | 2.32 | 13.35 | 0.00 |
| YRS | 3.25 | 1 | 9.59 | 2 | 8.82 | 0.00 | 6.06 | 1.16 | 3.34 | 0.00 |
| ZRB | 8.95 | 1 | 4.11 | 2 | 16.54 | 4.48 | 11.71 | 5.61 | 13.32 | 3.46 |
| ALX | 4.87 | 2 | 10.96 | 5 | 15.87 | 4.82 | 7.47 | 2.19 | 11.61 | 4.42 |
| ALT | 11.90 | 2 | 42.47 | 15 | 5.97 | 1.49 | 19.28 | 6.78 | 18.27 | 5.60 |
| DVS | 8.32 | 2 | 8.22 | 1 | 17.88 | 3.71 | 13.51 | 3.42 | 3.31 | 0.00 |
| GNT | 7.61 | 2 | 19.18 | 6 | 11.92 | 2.21 | 11.97 | 3.36 | 8.31 | 2.21 |
| JNS | 7.84 | 2 | 26.03 | 8 | 12.29 | 2.60 | 12.08 | 2.19 | 14.92 | 3.32 |
| KFB | 5.88 | 2 | 39.73 | 20 | 8.53 | 1.49 | 20.47 | 10.20 | 6.67 | 1.11 |
| MLR | 6.06 | 2 | 17.81 | 8 | 13.96 | 3.71 | 16.47 | 4.45 | 19.90 | 7.74 |
| OWN | 10.43 | 2 | 10.96 | 8 | 11.65 | 4.48 | 17.88 | 5.55 | 4.98 | 2.28 |
| WAT | 6.68 | 2 | 21.92 | 5 | 5.84 | 1.11 | 5.79 | 3.36 | 3.33 | 0.00 |
| WOD | 5.33 | 2 | 19.18 | 7 | 6.73 | 0.00 | 24.16 | 4.58 | 18.29 | 4.56 |
| BCK | 11.22 | 3 | 15.07 | 5 | 18.71 | 1.49 | 7.33 | 3.36 | 16.66 | 1.17 |
| BOU | 3.94 | 3 | 45.21 | 10 | 18.32 | 5.20 | 11.83 | 4.39 | 18.27 | 4.56 |
| BKL | 4.34 | 3 | 17.81 | 6 | 8.82 | 0.00 | 9.13 | 1.16 | 5.00 | 2.28 |
| DUB | 7.30 | 3 | 6.85 | 1 | 17.63 | 0.00 | 18.13 | 3.36 | 6.62 | 0.00 |
| GRB | 4.04 | 3 | 24.66 | 9 | 8.53 | 1.49 | 17.87 | 5.55 | 9.96 | 3.39 |
| GLB | 4.24 | 3 | 6.85 | 1 | 8.16 | 1.11 | 7.46 | 2.26 | 13.30 | 2.28 |
| HMN | 6.92 | 3 | 21.92 | 7 | 23.44 | 6.31 | 7.58 | 1.10 | 1.66 | 1.11 |
| LYN | 7.86 | 3 | 16.44 | 5 | 15.55 | 0.00 | 25.22 | 8.97 | 21.60 | 6.71 |
| PKT | 8.66 | 3 | 26.03 | 5 | 4.00 | 1.11 | 16.60 | 3.42 | 8.29 | 2.21 |
| RIN | 4.73 | 3 | 13.70 | 3 | 6.49 | 0.00 | 8.88 | 3.42 | 8.31 | 3.39 |
